# Flecainide in Structural Heart Disease: Reconsidering Its Role in Contemporary Arrhythmia Management

**DOI:** 10.3390/life16050778

**Published:** 2026-05-06

**Authors:** Paschalis Karakasis, Konstantinos Grigoriou, Panagiotis Theofilis, Panagiotis Iliakis, Panayotis K. Vlachakis, Nikolaos Ktenopoulos, Anastasios Apostolos, Dimitrios Patoulias, Antonios P. Antoniadis, Nikolaos Fragakis

**Affiliations:** 1 Second Department of Cardiology, Hippokration General Hospital, Medical School, Aristotle University of Thessaloniki, Konstantinoupoleos 49, 54642 Thessaloniki, Greece; aantoniadis@gmail.com (A.P.A.); fragakis.nikos@gmail.com (N.F.); 2 Department of Pharmacology, University of Athens, 75 Mikras Asias Avenue, 11527 Athens, Greece; dinosgrigoriou@gmail.com; 3 First Cardiology Department, School of Medicine, Hippokration General Hospital, National and Kapodistrian University of Athens, 11527 Athens, Greece; panos.theofilis@hotmail.com (P.T.); panayiotisiliakis@gmail.com (P.I.); vlachakispanag@gmail.com (P.K.V.); nikosktenop@gmail.com (N.K.); anastasisapostolos@gmail.com (A.A.); 4 Department of Medicine, Division of Cardiology, Angiology and Internal Emergency Medicine, Ruhr University Bochum, Knappschaft Kliniken University Hospital Bochum, 44892 Bochum, Germany; 5 Department of Cardiology, Guy’s and St Thomas’ NHS Foundation Trust, Harefield Hospital, London UB9 6JH, UK; patoulias@auth.gr; 6 Second Propedeutic Department of Internal Medicine, Faculty of Medicine, School of Health Sciences Aristotle, University of Thessaloniki, 54642 Thessaloniki, Greece

**Keywords:** flecainide, structural heart disease, atrial fibrillation, ventricular arrhythmias, coronary artery disease, arrhythmogenic right ventricular cardiomyopathy, premature ventricular complex-induced cardiomyopathy, class Ic antiarrhythmic drugs

## Abstract

Background: Flecainide has remained largely excluded from use in structural heart disease for more than three decades, mainly because of the Cardiac Arrhythmia Suppression Trial, which showed excess mortality in post-myocardial infarction patients treated for ventricular ectopy. However, the influence of this trial has extended well beyond the population actually studied, fostering a broad safety paradigm that may not fully reflect contemporary clinical practice. Aim: This review aims to re-examine the role of flecainide in structural heart disease by examining the historical basis for its restriction and contrasting it with emerging contemporary evidence across specific structural substrates. Discussion: Flecainide remains one of the most effective antiarrhythmic drugs for rhythm control in atrial fibrillation and for the suppression of selected ventricular arrhythmias in patients without overt structural abnormalities. Emerging observational and early prospective data suggest that, in carefully selected patients with stable coronary artery disease without active ischemia, preserved left ventricular function, arrhythmogenic right ventricular cardiomyopathy, and premature ventricular complex-induced cardiomyopathy, flecainide may provide meaningful antiarrhythmic benefit without a clear signal of excess proarrhythmia or mortality. Advances in cardiac imaging, ischemia assessment, and phenotypic risk stratification further support a more individualized approach to candidate selection. Conclusions: Flecainide should not be considered uniformly contraindicated across all forms of structural heart disease. Rather than supporting indiscriminate use, the available evidence supports a mechanistically informed and phenotype-specific reassessment of its role in selected patients. Prospective studies are needed to determine whether current guideline restrictions remain justified in the modern era.

*Key Messages*

- The historical restriction of flecainide in SHD is based largely on CAST and should not be extrapolated uncritically to all structural substrates.
- Flecainide-related risk appears to be substrate-dependent, with greatest concern in the presence of active ischemia, significant scar, reduced LV systolic function, or conduction disease.
- Contemporary evidence in selected phenotypes is encouraging but remains observational, retrospective, and highly selected; therefore, it should be regarded as hypothesis-generating rather than practice-changing.
- Any consideration of flecainide beyond conventional indications requires careful phenotyping, appropriate baseline evaluation, and close specialist monitoring.

## 1. Introduction

Flecainide occupies a paradoxical position in contemporary arrhythmia management. On one hand, it remains one of the most effective sodium channel blockers for rhythm control, with established roles in the pharmacological cardioversion of recent-onset atrial fibrillation (AF), the maintenance of sinus rhythm in symptomatic AF without significant structural heart disease (SHD), and the pill-in-the-pocket strategy in appropriately selected patients [1,2,3]. Beyond AF, current guideline-based practice also recognizes flecainide as a therapeutic option in selected supraventricular tachycardias, in symptomatic idiopathic premature ventricular complexes or ventricular tachycardia when catheter ablation is not feasible or desired, and as adjunctive therapy in catecholaminergic polymorphic ventricular tachycardia despite beta-blocker treatment [4,5,6].

Yet despite this broad and durable clinical utility, flecainide has remained largely excluded from structural heart disease for more than three decades. This caution derives overwhelmingly from the Cardiac Arrhythmia Suppression (CAST) Trial [7,8], which demonstrated excess arrhythmic and all-cause mortality in post-myocardial infarction patients with ventricular ectopy treated with class Ic agents. Although the trial’s findings remain indisputable, their influence has extended far beyond the ischemic, recently infarcted, and often incompletely revascularized population actually studied, shaping a safety paradigm that has frequently been generalized to disparate substrates such as stable coronary artery disease, hypertrophic remodeling, nonischemic cardiomyopathy, valvular disease, congenital heart disease, and arrhythmogenic cardiomyopathy.

**Figure 1:**
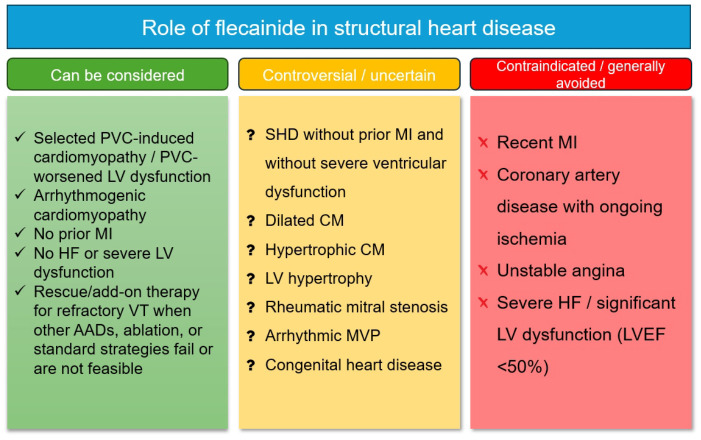
Proposed contemporary role of flecainide in structural heart disease. Abbreviations: AAD, anti-arrhythmic drug; CM, cardiomyopathy; HF, heart failure; LV, left ventricular; LVEF, left ventricular ejection fraction; MI, myocardial infarction; MVP, mitral valve prolapse; PVC, premature ventricular complex; SHD, structural heart disease; VT, ventricular tachycardia.

That broad exclusion is increasingly difficult to reconcile with contemporary evidence. Advances in cardiac imaging, ischemia testing, phenotypic risk stratification, and deeper mechanistic understanding of arrhythmogenic remodeling—including molecular and epigenetic pathways—have fundamentally changed how arrhythmic substrate is conceptualized and classified [9,10,11,12]. At the same time, emerging observational and early prospective data raise the possibility that, in carefully selected patients, flecainide may have a more nuanced risk–benefit profile than that implied by broad categorical exclusion [13]. This is especially relevant at a time when precision rhythm control is increasingly emphasized, yet therapeutic options remain limited once structural heart disease is invoked as a blanket contraindication. However, data remain preliminary, largely nonrandomized, and insufficient to support broad use in structural heart disease. Against this background, a critical reappraisal of flecainide is warranted—not to relax safety principles indiscriminately, but to determine whether a decades-old prohibition has, in some settings, outlived the evidence on which it was built (Figure 1).

## 2. Pharmacodynamic and Pharmacokinetic Properties of Flecainide

Flecainide acetate is a class IC antiarrhythmic agent whose principal pharmacologic action is strong use- and voltage-dependent inhibition of the fast sodium current mediated by Nav1.5 channels in atrial and ventricular myocardium, as well as in the His–Purkinje conduction system [14]. Through attenuation of the phase 0 action potential upstroke, the drug produces pronounced slowing of impulse propagation, which explains its marked dromotropic effects, while having comparatively little effect on ventricular repolarization [15,16,17,18]. In addition to its impact on conduction, flecainide increases refractoriness within cardiac tissue, thereby reinforcing its antiarrhythmic activity [19,20,21]. Although blockade of sodium channels represents its dominant mechanism, flecainide also interacts with other ionic pathways, including inhibition of the rapid delayed rectifier and transient outward potassium currents [19,20,21]. Experimental evidence has further indicated that the drug may influence intracellular calcium homeostasis by inhibiting ryanodine receptor type 2 channels, a mechanism that could reduce spontaneous calcium release from the sarcoplasmic reticulum and thereby limit triggered arrhythmogenesis [22].

The electrophysiologic actions of flecainide are mirrored by a characteristic electrocardiographic pattern, most notably PR interval prolongation and QRS complex widening, whereas effects on QT duration are generally modest. The degree of these electrocardiographic changes is not uniform and may be shaped by interindividual variability, pre-existing conduction abnormalities, and systemic drug exposure [15]. From a clinical perspective, another important property of flecainide is its negative inotropic action, which appears to increase in parallel with the extent of QRS prolongation [23]. This effect carries particular relevance in patients with HF or established LV systolic dysfunction, in whom further depression of myocardial contractile reserve may precipitate or worsen hemodynamic deterioration [24,25].

After oral administration, flecainide is absorbed rapidly and extensively, achieving a bioavailability in the range of 90% to 95%, with peak plasma concentrations typically observed approximately 3 h after ingestion [26]. Protein binding in plasma is moderate, generally reported between 32% and 58%. The drug is metabolized predominantly in the liver via cytochrome P450 2D6, with a lesser contribution from cytochrome P450 1A2, and both the parent compound and its metabolites are eliminated mainly through renal excretion, with a smaller fraction recovered in the feces. In individuals without significant renal or hepatic impairment, the elimination half-life is approximately 13 h [26]. These pharmacokinetic features have direct clinical relevance because flecainide possesses a narrow therapeutic index [27]. Consequently, reductions in renal or hepatic clearance may favor drug accumulation and increase susceptibility to dose-related toxicity, highlighting the need for individualized dose selection and vigilant monitoring in patients with impaired organ function [28].

## 3. CAST and the Overextension of Its Findings to Structural Heart Disease

The CAST tested whether suppression of premature ventricular complexes after myocardial infarction could reduce arrhythmic death [7,8]. Between 6 days and 2 years after myocardial infarction, 1498 patients with at least 6 premature ventricular complexes per hour on Holter monitoring were enrolled; 755 were assigned to encainide or flecainide and 743 to placebo. The trial was terminated early after a mean follow-up of 10 months because active treatment was associated with a marked excess of harm, including a 3.6-fold higher risk of arrhythmic death (95% confidence interval 1.7–7.9) and a 2.5-fold higher risk of total mortality (95% confidence interval 1.6–4.5). These findings justifiably led to a boxed warning against flecainide in patients with prior myocardial infarction and reduced left ventricular ejection fraction. However, the subsequent extension of these results to structural heart disease more broadly has often exceeded the actual evidentiary scope of the trial [1,4].

A major interpretive error was the extrapolation of CAST from a narrowly defined post-infarction population to heterogeneous forms of structural heart disease, including cardiomyopathies [29], left ventricular hypertrophy, and stable coronary artery disease, despite the absence of direct supportive data [30]. This broad generalization overlooked the fact that CAST enrolled a distinctly high-risk cohort: most patients entered the study within 30 days of myocardial infarction (78.6% in the active-treatment group and 78.1% in the placebo group), left ventricular systolic dysfunction was common, and only 21.0% and 18.6%, respectively, had a left ventricular ejection fraction >50%. Moreover, revascularization was incomplete by contemporary standards. Thrombolysis was used in 28.2% of the active-treatment group and 24.3% of the placebo group, whereas percutaneous coronary intervention was performed in only 19.1% and 18.7%, and coronary artery bypass grafting in 18.6% of both groups. Consequently, 34.1% of patients receiving active therapy and 38.4% of those receiving placebo underwent no revascularization at all, suggesting that residual ischemia remained prevalent.

CAST also targeted suppression of asymptomatic premature ventricular complexes, rather than treatment of clinically significant arrhythmias. This is critical, because the risk–benefit balance of antiarrhythmic therapy depends heavily on the arrhythmia being treated and the clinical context in which treatment is given. In a population with recent myocardial infarction, impaired ventricular function, and likely ongoing ischemia, exposing patients to class IC therapy for suppression of a surrogate marker may have created conditions in which proarrhythmia outweighed any plausible clinical benefit [31]. Mechanistically, this is biologically coherent: flecainide-induced conduction slowing within an ischemic and electrically heterogeneous substrate may facilitate re-entry and promote ventricular arrhythmias [30,32,33,34,35]. Consistent with this concept, CAST subanalyses identified non-Q-wave myocardial infarction as a strong predictor of arrhythmic events and all-cause mortality in patients treated with encainide or flecainide, underscoring the likelihood that residual ischemia, rather than structural heart disease per se, was a major determinant of adverse outcome [34,35].

From a mechanistic perspective, the arrhythmic effect of flecainide is likely to differ between active ischemic tissue and chronic fibrotic remodeling. In acute or ongoing ischemia, membrane depolarization, reduced sodium channel availability, acidosis [36,37], and impaired intercellular and ephaptic coupling may accentuate flecainide-induced slowing of impulse propagation [38,39], thereby increasing the likelihood of conduction block and re-entry in an already heterogeneous substrate. In contrast, in chronic fibrotic myocardium, arrhythmic risk is more likely to depend on scar architecture, anisotropic conduction, source–sink mismatch, and the remaining conduction reserve, rather than on ischemia-driven sodium channel vulnerability alone [40,41,42,43]. This helps explain why the proarrhythmic signal observed in CAST should not necessarily be extrapolated uniformly across all structural phenotypes.

Accordingly, CAST should be understood as a landmark warning against class IC therapy in patients with recent myocardial infarction, left ventricular dysfunction, and incompletely stabilized ischemic substrate, not as definitive evidence that flecainide is uniformly unsafe across all forms of structural heart disease. The findings remain valid; their indiscriminate extension does not.

## 4. The Changing Landscape of Coronary Artery Disease Since CAST

What has changed since CAST is not the validity of its central safety signal, but the clinical landscape in which that signal is interpreted. Coronary artery disease is no longer viewed as a uniform entity, and contemporary patients with stable coronary disease differ substantially from the high-risk post-myocardial infarction population enrolled in CAST [44,45]. In particular, stable nonobstructive coronary artery disease, preserved left ventricular systolic function, absence of residual ischemia, widespread use of contemporary secondary prevention, and far more complete revascularization define a markedly different arrhythmic substrate from that of patients with recent infarction and incompletely treated ischemic disease [46]. This distinction is clinically important because the proarrhythmic risk associated with flecainide is likely driven less by the mere presence of coronary atherosclerosis than by the presence of active ischemia, recent infarction, ventricular dysfunction, and electrically unstable scar-related substrate. Accordingly, an important contemporary challenge is to move beyond the overly broad concept of “any coronary artery disease” as an absolute contraindication and toward a more nuanced definition of coronary disease that is genuinely relevant to proarrhythmic risk. From this perspective, CAST remains highly informative, but its conclusions should be applied to the specific ischemic phenotype it studied rather than indiscriminately extended to all forms of coronary artery disease or structural heart disease. An additional distinction of practical importance is whether prior infarction has been followed by true substrate stabilization. A patient with remote myocardial infarction, complete revascularization, no residual or inducible ischemia, preserved left ventricular systolic function, and no significant scar on cardiac magnetic resonance imaging should not be assumed to carry the same proarrhythmic substrate as the CAST population, which was characterized by recent infarction, frequent ventricular ectopy, ventricular dysfunction, incomplete contemporary revascularization, and likely prevalent residual ischemia. In this respect, revascularization status is not merely an anatomic descriptor, but a marker of ongoing arrhythmic vulnerability, as incomplete revascularization after myocardial infarction has been associated with higher long-term arrhythmic risk than complete revascularization [47]. Nevertheless, complete revascularization does not in itself eliminate risk, because infarct-related fibrosis and peri-infarct heterogeneity may persist even in the absence of active ischemia. Accordingly, the relevant contemporary distinction is not simply between obstructive and nonobstructive coronary disease, but between active or incompletely stabilized ischemic substrate and remote, stabilized substrate defined by ischemia status, revascularization completeness, ventricular function, and scar burden/pattern. Even so, prospective flecainide-specific data remain insufficient in patients with remote revascularized infarction, and this phenotype should therefore be regarded as conceptually lower risk than CAST-like patients, but not yet definitively established as safe.

## 5. Emerging Evidence for Use of Flecainide in Structural Heart Disease

In the present review, structural heart disease is not treated as a single homogeneous entity, but as an umbrella term encompassing distinct substrates with potentially different proarrhythmic implications for flecainide therapy. For interpretive purposes, we distinguish (i) ischemic from nonischemic structural disease; (ii) active or incompletely stabilized ischemic substrates—such as recent myocardial infarction, residual or inducible ischemia, incomplete revascularization, or unstable scar-related substrate—from remote, stabilized coronary disease; (iii) preserved from reduced left ventricular systolic function, using a conservative practical threshold of LVEF < 50% as a marker of heightened concern in this review; and (iv) the absence versus presence of myocardial scar/fibrosis, particularly when identified by cardiovascular magnetic resonance with late gadolinium enhancement (LGE). Within this framework, flecainide-related risk is viewed as substrate-dependent rather than diagnosis-dependent.

### 5.1. Coronary Artery Disease

The evidence base in coronary artery disease is increasingly difficult to reconcile with the blanket post-CAST prohibition of flecainide (Table 1). Although CAST established clear harm in patients with recent myocardial infarction, frequent ventricular ectopy, and impaired left ventricular function, more contemporary datasets suggest that this risk does not uniformly extend to all patients with coronary artery disease. Rather, the safety signal appears to depend on the substrate: recent infarction, residual ischemia, unrevascularized obstructive disease, and ventricular dysfunction remain the major concerns, whereas selected patients with stable or revascularized coronary artery disease and preserved ventricular function may represent a fundamentally different population [46,48].

Several observational studies support this more selective view. In the Mayo Clinic series of 348 patients treated with flecainide for at least 1 year [46], 10-year survival was similar among those with no/minimal, nonobstructive, and obstructive coronary artery disease (*p* = 0.6), with no significant difference in arrhythmia burden, including sustained ventricular tachycardia or frequent premature ventricular complexes (*p* = 0.25). Even among patients with reversible perfusion defects on myocardial perfusion imaging, mortality was not increased (*p* = 0.14), and no signal emerged for multivessel disease (*p* = 0.89).

The largest real-world analysis, however, tempers excessive optimism. In the Emory cohort [50], which included 3445 class Ic-treated patients and 2216 class III-treated controls, class Ic therapy was not associated with excess mortality overall after adjustment, but outcomes differed by coronary phenotype: in patients with obstructive coronary artery disease, class Ic use was associated with poorer event-free survival compared with sotalol (hazard ratio 3.80, 95% confidence interval 1.67–8.67; *p* = 0.002). By contrast, among selected patients with nonobstructive coronary artery disease and no history of ventricular tachycardia, no increase in mortality was observed [50]. These data suggest that the key distinction is not simply the presence of coronary atherosclerosis, but whether the substrate is low-risk and stable versus obstructive and potentially proarrhythmic.

Additional contemporary studies further challenge the notion that flecainide is uniformly hazardous in coronary disease. In the analysis by Burnham et al. [51], propensity-matched patients with atrial fibrillation and stable coronary artery disease treated with flecainide (n = 1114) had lower 3-year mortality than those receiving class III antiarrhythmic drugs (9.1% vs. 19.3%, *p* < 0.0001), along with lower rates of heart failure hospitalization (12.5% vs. 18.3%, *p* < 0.0001), major adverse cardiovascular events (22.9% vs. 36.6%, *p* < 0.0001), and ventricular tachycardia (5.8% vs. 8.5%, *p* = 0.02). In the subgroup with prior percutaneous coronary intervention or coronary artery bypass grafting, outcomes again numerically favored flecainide, although differences were not statistically significant. Similarly, in the nationwide Taiwan cohort of 3750 patients with new-onset atrial fibrillation after percutaneous coronary intervention [52], class Ic therapy was associated with lower risks of major adverse cardiovascular events (adjusted subdistribution hazard ratio 0.64, 95% confidence interval 0.59–0.68), all-cause mortality (0.61, 95% confidence interval 0.57–0.66), and cerebrovascular events (0.81, 95% confidence interval 0.66–0.99), without an increase in ventricular arrhythmia (0.89, 95% confidence interval 0.69–1.15; *p* = 0.37).

Prospective data, although still limited, are directionally consistent. In EAST-AFNET 4 [48], flecainide or propafenone was used in 689 patients, including 41 with prior myocardial infarction, coronary artery bypass grafting, or percutaneous coronary intervention, with a median treatment duration of 1153 days. The primary efficacy outcome occurred less often during sodium channel blocker therapy than in patients never receiving these agents (3.0 vs. 4.9 per 100 patient-years; *p* < 0.001), while primary safety outcomes were numerically lower (2.9 vs. 4.2 per 100 patient-years; adjusted *p* = 0.015). Sinus rhythm at 2 years was maintained in 88% versus 82%. Preliminary randomized data from FLECA-ED [53,55] extend this concept to the acute setting: among patients with coronary artery disease, preserved ejection fraction, and no residual ischemia, intravenous flecainide achieved a median time to cardioversion of 35 min versus 679 min with amiodarone (*p* < 0.001), and 10 of 10 patients (100%) in the flecainide arm were discharged from the emergency department in sinus rhythm within 6 h, whereas 11 of 15 (73%) treated with amiodarone required hospitalization.

### 5.2. Left Ventricular Hypertrophy

The evidence base for flecainide in left ventricular hypertrophy (LVH) is limited, but the available data do not support a uniform assumption of excess proarrhythmia in this setting.

The most directly relevant data come from the recent retrospective cohort by Sangpornsuk et al. [54], in which 47 of 336 patients (13.9%) had structural heart disease and 13 of these 47 (28%) had LV wall thickness ≥ 14 mm. Across the entire structural heart disease cohort, VT/VF occurred in 4.2% versus 1.0% in patients without structural heart disease, but this difference was not statistically significant (*p* = 0.17), and structural heart disease was not independently associated with ventricular arrhythmia after adjustment (OR 4.8, 95% CI 0.6–38.44; *p* = 0.14) [54]. Crucially, the authors specifically note that no VT was reported in the LVH subgroup, despite current guideline-based concern regarding flecainide in this setting. Their cohort was admittedly low-risk—maximum LV wall thickness of 16 mm and minimum LVEF of 35%—but the signal is nonetheless reassuring.

Additional support comes from the retrospective study by Haruki et al. [56], in which 15 patients with hypertrophic obstructive cardiomyopathy and a mean LV wall thickness of 20.4 mm were treated with flecainide and followed for 8.9 years, with no malignant arrhythmias detected. Although small and nonrandomized, this is notable because it extends the safety signal beyond mild hypertrophy into a phenotype traditionally viewed as particularly vulnerable [56]. Likewise, the broader structural heart cohort in Sangpornsuk suggests that once major ischemic disease and advanced ventricular dysfunction are excluded, flecainide-related ventricular toxicity may be less common than conventionally assumed.

Kirchhof’s Flec-SL trial [49] adds only indirect support and should be interpreted cautiously in this context, since most participants had little or no structural heart disease; mean diastolic septal and posterior wall thicknesses were only 11.3 mm and 11.2 mm, respectively. Still, it is relevant that flecainide was effective and that serious adverse events remained uncommon in a population with largely preserved cardiac structure.

### 5.3. Valvular Heart Disease

The evidence for flecainide in valvular heart disease is limited, but the available data are more encouraging than traditional guideline caution would suggest (Table 2). Importantly, the relevant contemporary cohorts differ fundamentally from CAST: they largely involve younger patients, preserved ventricular function, and nonischemic valvular substrates, rather than post-myocardial infarction scar. In this context, flecainide has shown both meaningful rhythm-control efficacy and a reassuring short- to mid-term safety profile in selected patients with rheumatic and mitral valve-related disease.

The most substantive data come from rheumatic valvular AF after mitral intervention. In the pilot study by Ghosh et al. [57], which included 50 patients with rheumatic AF after successful balloon mitral valvotomy, a combined strategy of oral flecainide plus cardioversion restored sinus rhythm in 38 of 50 patients (76%) at discharge; 30 of 38 initial converters (79%) and 30 of 50 overall patients (60%) remained in sinus rhythm at 1 year [57]. Flecainide was well tolerated, with no major complications, no systemic embolism, no deaths, and no proarrhythmic events during follow-up. Patients who maintained sinus rhythm also had better functional status and quality of life, with lower New York Heart Association class (1.1 ± 0.12 vs. 1.3 ± 0.10; *p* = 0.03) and higher physical quality-of-life scores (50.11 ± 5.34 vs. 46.84 ± 5.38; *p* = 0.02) [57].

A similar signal was reported by Tripathi et al. [58] in 25 patients with chronic rheumatic AF after mitral valve replacement. A single oral flecainide dose restored sinus rhythm in 6 of 25 patients (24%), and with adjunctive direct-current cardioversion 21 of 25 (84%) were discharged in sinus rhythm. At 6 months, sinus rhythm was maintained in 16 of 21 initial converters (76%) and in 16 of 25 overall patients (64%). Safety outcomes were again reassuring: no systemic thromboembolism, no major bleeding, no hospitalizations, and no deaths were observed, and there were no significant changes in PR interval, QRS duration, or corrected QT interval overall [58]. Patients remaining in sinus rhythm had better functional status (1.1 ± 0.12 vs. 1.4 ± 0.10; *p* = 0.03), better quality-of-life scores (55.88 ± 8.37 vs. 62.43 ± 12.25; *p* = 0.014), and more favorable left atrial strain (25.25 ± 2.59% vs. 17.43 ± 1.9%; *p* < 0.0001) [58].

Beyond rheumatic disease, flecainide may also have a role in arrhythmic mitral valve phenotypes. In the small case series by Aabel et al. [59], 7 high-risk patients with arrhythmic mitral valve syndrome refractory to beta-blockers received flecainide in addition to low-dose beta-blockade. Over 250 patient-months of treatment, 0 nonsustained ventricular tachycardia episodes were observed, compared with 21 episodes during 90 patient-months on beta-blocker alone [59]. Premature ventricular complex burden fell from a median of 4.2% per 24 h to 0.4% per 24 h, corresponding to a reduction of −3.4% per 24 h (95% CI −5.0 to −1.8; *p* < 0.001) [59]. No serious adverse effects were reported, although QRS duration increased modestly by 16 ms on treatment [59].

### 5.4. Nonischemic Cardiomyopathy

#### 5.4.1. Dilated Cardiomyopathy

Data on flecainide in dilated/nonischemic cardiomyopathy remain limited, but the emerging signal is more reassuring than conventional dogma would suggest, particularly when ischemia has been excluded and arrhythmic protection is enhanced by an implantable cardioverter–defibrillator (Table 3). In the most informative contemporary cohort, Raad et al. [60] studied 34 patients with nonischemic cardiomyopathy and implantable cardioverter–defibrillators, of whom 23 received flecainide. Treatment reduced premature ventricular complex burden from 20% ± 13% to 6% ± 7% (*p* < 0.001), increased left ventricular ejection fraction from 33% ± 9% to 37% ± 10% (*p* = 0.01), and improved biventricular pacing from 85% ± 9% to 93% ± 7% (*p* = 0.005) [60]. Importantly, safety outcomes were not adverse: sustained ventricular tachycardia occurred in only 2 patients during therapy versus 9 in the 12 months before treatment, heart failure admissions reduced from 3 to 2, and no deaths occurred during follow-up [60].

A larger real-world dataset by Sherman et al. [61] extends this safety signal. Among 210 patients with nonischemic cardiomyopathy treated with class Ic agents (86 flecainide, 124 propafenone) for a mean of 4.34 years, no sustained ventricular tachycardia and no cardiac deaths were observed during treatment. Only 9 patients (4.29%) had nonsustained ventricular tachycardia, and although 5 deaths (2.3%) occurred, all were adjudicated as unrelated to antiarrhythmic therapy [61].

#### 5.4.2. Arrhythmogenic Cardiomyopathy

Among the structural heart disease phenotypes in which flecainide has been re-evaluated most convincingly, arrhythmogenic cardiomyopathy, particularly arrhythmogenic right ventricular cardiomyopathy, stands out. The strongest contemporary evidence comes from the multicenter study by Gaine et al. [62], which included 191 patients with definite arrhythmogenic right ventricular cardiomyopathy, 59.2% of whom had an implantable cardioverter–defibrillator, 33.0% prior sustained ventricular arrhythmia, and 34.6% left ventricular involvement. Over a median on-drug follow-up of 4.2 years, flecainide was well tolerated, with a discontinuation rate of only 7.9%. Treatment was associated with a marked reduction in arrhythmic burden, with 24 h premature ventricular complex burden falling from 2190 to 418 (*p* < 0.001) and nonsustained ventricular tachycardia decreasing from 35.1% to 21.5% (*p* = 0.003) [62]. In those with prior ventricular arrhythmia, the yearly event rate fell from 1.1 [0.4–1.6] episodes/year to 0 [0–0.3] episodes/year (*p* < 0.001) [62]. Importantly, these benefits were consistent regardless of genotype and were also observed in patients with left ventricular involvement [62].

Earlier single-center studies point in the same direction. In the cohort reported by Rolland et al. [63], 100 consecutive patients with arrhythmogenic right ventricular cardiomyopathy received flecainide in combination with beta-blockers [63]. Premature ventricular complex burden on Holter decreased from a median of 2370 [1572–3400] to 415 [97–730] (*p* < 0.0001), while programmed ventricular stimulation positivity fell from 94% off treatment to 40% on treatment (*p* < 0.001) [63]. During a median follow-up of 47 months, sustained ventricular arrhythmia occurred in 22 patients, corresponding to an event rate of 5% at 1 year and 25% at 5 years, with no deaths and no reported Brugada pattern or hemodynamic deterioration [63].

Supportive mechanistic–clinical evidence also comes from the smaller series by Ermakov et al. [64], in which 8 patients with arrhythmogenic right ventricular cardiomyopathy refractory to single-agent therapy and/or ablation were treated with flecainide plus sotalol or metoprolol. Six of the eight achieved excellent arrhythmia control and remained arrhythmia-free for a mean of 35.5 months, whereas the two failures recurred within 2 months and proceeded to repeat ablation [64].

#### 5.4.3. Premature Ventricular Complex-Induced Cardiomyopathy

Perhaps the strongest mechanistic rationale for flecainide in structural heart disease is in PVC-induced cardiomyopathy, where the arrhythmia is not merely a marker of disease but a driver of ventricular dysfunction. In the cohort reported by Hyman et al. [65], 20 patients with suspected PVC-induced cardiomyopathy, most of whom had already failed ablation (1.3 ± 0.2 prior unsuccessful procedures), were treated with flecainide or propafenone. Mean PVC burden fell from 36.2% ± 3.5% to 10.0% ± 2.4% (*p* < 0.001), accompanied by an increase in left ventricular ejection fraction from 37.4% ± 2.0% to 49.0% ± 1.9% (*p* < 0.001) [65]. Even among the subgroup with myocardial delayed enhancement on cardiac magnetic resonance imaging, left ventricular ejection fraction improved from 36.8% ± 4.3% to 51.7% ± 3.7% (*p* < 0.01). Over 3.8 ± 0.9 treatment-years, no sustained ventricular arrhythmias or sudden cardiac deaths occurred [65].

This concept is also supported by gene-mediated arrhythmic dilated cardiomyopathy caused by the p.R222Q SCN5A variant, which behaves as a highly arrhythmogenic, sodium channel-mediated form of PVC-driven ventricular dysfunction. In the original family study by Mann et al. [66], sodium channel-blocking therapy with flecainide or amiodarone produced a dramatic reduction in ventricular ectopy together with recovery of left ventricular function in affected carriers with cardiomyopathy. Long-term follow-up by Peters et al. [67] further reinforced this signal: among 8 treated family members followed for 1 to 15 years (mean 8 years), 7 had multifocal ventricular ectopy (3% to 58%) and dilated cardiomyopathy at baseline, and in each case sodium channel blockade reduced ventricular ectopy to <1% and normalized systolic function. No new ventricular arrhythmic complications occurred in flecainide-treated patients [67].

Taken together, these data indicate that when ventricular dysfunction is driven predominantly by ectopic burden, flecainide can do more than suppress arrhythmia; it can facilitate reverse remodeling. Although the evidence remains observational and highly selected, it provides some of the clearest support for reconsidering flecainide in carefully phenotyped nonischemic substrates.

### 5.5. Congenital Heart Disease

The evidence for flecainide in congenital heart disease (CHD) is derived mainly from pediatric cohorts, but overall it is more reassuring than older extrapolations from CAST would suggest (Table 4). In the contemporary 2-center study by Cunningham et al. [69], 175 children received flecainide, including 20 with CHD and 2 with cardiomyopathy. Arrhythmia control was achieved in 90% of patients with CHD compared with 77% in those with normal hearts, with no significant difference between groups. Importantly, serious safety events were uncommon: flecainide-associated cardiac dysfunction requiring discontinuation occurred in only 2 patients (1%), proarrhythmia in 3 patients (2%), and no cardiac arrests were observed during follow-up. Even though severe baseline ventricular dysfunction was more frequent in the CHD group (30% vs. 8%), adverse event rates were not higher [69].

These findings are supported by the larger multicenter administrative analysis by Moffett et al. [74], which included 3544 children with CHD or cardiomyopathy receiving antiarrhythmic therapy for supraventricular arrhythmias, of whom 229 (6.5%) received flecainide [74]. In this higher-risk structural cohort, the incidence of cardiac arrest with flecainide was 3.0% and overall mortality 4.3%; however, on multivariable analysis, flecainide was not associated with a higher risk of cardiac arrest or death compared with other antiarrhythmic agents [74]. Notably, mortality in cardiomyopathy patients treated with flecainide was 2.9%, and no patient with single-ventricle physiology died [74].

Analyzed together, these data suggest that flecainide should not be viewed as uniformly prohibitive in congenital heart disease. In carefully selected patients, particularly when ventricular function and conduction are monitored closely, it appears to offer meaningful antiarrhythmic efficacy without a clear excess of major safety events relative to alternative drug therapy.

## 6. Contradictions and Percussions

In routine clinical practice, flecainide should generally be avoided in patients with significant baseline conduction delay, particularly when the QRS duration exceeds 120 ms or when left bundle branch block or bifascicular block is present [2,75,76]. Its use is likewise discouraged in the setting of coronary artery disease, heart failure, cardiogenic shock, or reduced left ventricular ejection fraction, and it remains contraindicated in Brugada syndrome. By contrast, an incidental Agatston calcium score <400 in an asymptomatic patient, as well as uncomplicated left ventricular hypertrophy in the absence of myocardial scar, does not necessarily preclude treatment [2]. Because flecainide undergoes substantial renal elimination, impaired kidney function—especially a glomerular filtration rate <35 mL/min—should prompt either avoidance or dose reduction. Unless pacing backup is available, flecainide is also not recommended in patients with sinus node dysfunction, atrial conduction disease, second-degree or higher atrioventricular block, or bundle branch block. As with any drug, hypersensitivity constitutes an absolute contraindication. It should also be acknowledged that the definition of preserved left ventricular function is not uniform across the available literature, with different studies using different LVEF thresholds, in some cases >35%, >40%, or >50%. This heterogeneity largely reflects the broad time span of the published evidence and the fact that many studies predate more contemporary ESC-based conceptual frameworks. In the present review, we deliberately adopt a more conservative interpretive threshold; accordingly, as illustrated in Figure 1, flecainide is considered contraindicated in patients with LVEF < 50%, rather than relying on the lower cutoffs used in some earlier observational studies.

In patients with AF, flecainide may be combined with β-blockers or non-dihydropyridine calcium channel blockers such as verapamil or diltiazem to reduce the risk of rapid ventricular response during recurrent arrhythmia or in the event of organization into type Ic atrial flutter [2]. Particular caution is required when flecainide is prescribed with renally cleared β-blockers, such as atenolol, because concomitant impairment of renal function may promote drug accumulation, toxicity, and profound bradycardia [2]. Patients should also be advised to avoid physical exertion during breakthrough arrhythmic episodes until sinus rhythm has been restored spontaneously or by active cardioversion.

From a practical standpoint, a tailored approach to flecainide use in structural heart disease also requires structured monitoring. Patients considered for treatment should undergo a baseline 12-lead ECG, with reassessment after drug initiation or dose escalation once steady-state exposure has been achieved, and periodic follow-up thereafter, with particular attention to PR interval, QRS duration, and the emergence of conduction abnormalities. Because flecainide exhibits use-dependent sodium channel blockade, exercise testing may also be considered in selected patients to detect excessive rate-related QRS widening or exercise-induced ventricular arrhythmias, particularly when latent ischemia or limited conduction reserve is a concern. This is especially relevant in patients with structural substrates in whom proarrhythmic risk may not be fully apparent under resting conditions.

## 7. Evidence Gaps and Future Directions

Future progress in the field will depend on moving beyond binary labels such as “structural heart disease” toward a more substrate-centered framework for flecainide use. Current European [1,4] and American [77,78,79] recommendations continue to treat CAD and most forms of SHD as broad contraindication categories, despite growing evidence that proarrhythmic risk is not dictated by diagnosis alone, but by the nature, extent, and activity of the underlying arrhythmogenic substrate. The next generation of guidance should therefore distinguish between phenotypes with active ischemia, infarct-related scar, advanced ventricular dysfunction, or extensive fibrosis [80] and those with more stable or functionally limited substrates, such as selected patients with nonobstructive or revascularized CAD, mild LVH, or ectopy-driven cardiomyopathy. Such a shift would align flecainide prescribing with contemporary disease characterization, in which risk is increasingly defined by ischemia burden, scar distribution, ventricular function, and electrophysiological vulnerability rather than by a broad diagnostic label alone [44,81].

A central research priority is the prospective definition of the appropriate candidate for flecainide in SHD. This will require studies that integrate clinical variables with multimodality imaging, particularly cardiovascular magnetic resonance for scar detection and tissue characterization, ischemia testing, electrocardiographic indices of conduction reserve, and, where relevant, genotype-informed phenotyping [82,83,84,85]. Cardiac magnetic resonance with LGE deserves particular emphasis within this substrate-based framework, because it permits direct visualization of replacement fibrosis and scar rather than relying solely on diagnostic labels or global ventricular function [86]. In principle, the absence of LGE is the most reassuring imaging scenario, whereas the presence of extensive or high-risk scar patterns should prompt greater caution. However, a universally “safe” LGE threshold for flecainide use cannot currently be defined. Although some disease-specific studies—most notably in hypertrophic cardiomyopathy—have proposed quantitative thresholds for extensive fibrosis, these were derived for arrhythmic risk stratification rather than for class Ic drug eligibility, and they vary substantially according to phenotype and quantification method. Accordingly, minimal LGE should not be equated with proven safety, and LGE findings should instead be integrated with LVEF, ischemia status, scar pattern, and overall conduction reserve when considering flecainide in structural heart disease. Rather than asking whether flecainide is safe in SHD as a whole, future investigations should determine in which specific substrates, and at which thresholds of LVEF, LVH, scar burden, or CAD severity, the benefit–risk ratio becomes acceptable. The same principle applies across disease categories: although ARVC and post-MI patients may share re-entry-based mechanisms and monomorphic ventricular tachycardia as a clinical phenotype, their underlying myocardial substrates differ fundamentally, and these mechanistic distinctions should inform therapeutic decision-making [84].

At the same time, the current evidence base remains dominated by small- to medium-sized observational studies, leaving substantial uncertainty regarding both safety and comparative efficacy. An important limitation of the contemporary literature is potential prescriber bias. Because flecainide is likely to be prescribed preferentially to patients with SHD who have preserved ventricular function, no active ischemia, lower scar burden, and an overall more favorable substrate, observational studies may overestimate its safety by selectively capturing lower-risk patients. This channeling effect limits causal inference and highlights the need for prospective studies with rigorous phenotyping and clearer control of baseline risk. In addition, some of the more reassuring safety signals may reflect not only selective prescribing to lower-risk substrates, but also the presence of protective contextual factors within the studied cohorts, including implantable cardioverter–defibrillator (ICD) backup, complete revascularization, exclusion of active ischemia, and close specialist monitoring, all of which may limit generalizability to routine real-world practice. Pragmatic multicenter registries and targeted randomized trials are therefore needed to validate the encouraging signals observed in stable CAD, ARVC, PVC-induced cardiomyopathy, selected nonischemic cardiomyopathies, and valvular phenotypes [46,53,67]. These studies should not be limited to hard outcomes such as mortality or sustained ventricular arrhythmia, but should also include arrhythmia burden, hospitalization, quality of life, reverse remodeling, and treatment discontinuation. Because flecainide is an off-patent drug, conventional industry-sponsored trial models may be difficult to realize; accordingly, academic collaborative networks and registry-based randomized designs may offer the most realistic path forward.

Finally, even if future evidence supports broader use of flecainide in selected SHD phenotypes, implementation will still require a disciplined monitoring strategy. Risk is dynamic rather than static: a patient with stable CAD may subsequently develop acute ischemia, a presumed PVC-cardiomyopathy may later prove to reflect a broader myocardial disease, and conduction reserve may deteriorate over time under the influence of age, comorbidity, electrolyte imbalance, renal dysfunction, or drug interactions [87,88]. For this reason, flecainide in emerging SHD indications should remain the domain of careful specialist oversight, with periodic reassessment of substrate stability, conduction parameters, organ function, and competing therapeutic options. The future of flecainide in SHD is therefore unlikely to lie in universal liberalization, but in a precision-based model of use grounded in mechanistic phenotyping, rigorous follow-up, and shared decision-making [89].

## 8. Conclusions

Flecainide should not be viewed as having a uniform risk profile across all SHD phenotypes; however, the current evidence base remains insufficient to justify broad clinical use beyond the established indications. The most concerning substrates continue to be recent myocardial infarction, active ischemia, significant scar, and advanced ventricular dysfunction, in keeping with existing guideline restrictions. Although selected observational studies suggest that some carefully phenotyped populations—such as patients with stable coronary disease without active ischemia, arrhythmogenic cardiomyopathy, or PVC-induced cardiomyopathy—may merit further investigation, these signals remain hypothesis-generating and are vulnerable to selection bias and residual confounding. Accordingly, current guideline restrictions remain valid in routine clinical practice, and the principal implication of the present review is not that flecainide is generally safe in SHD, but that prospective phenotype-specific studies are needed to determine whether some currently excluded subgroups may ultimately prove appropriate candidates under specialized care.

## Figures and Tables

**Table 1:** Studies Evaluating the Efficacy and Safety of Flecainide in Coronary Artery Disease.

Study/Author	Study Design	Population Included	Number of Patients	Findings (Efficacy and Safety)
Kirchhof et al. (Flec-SL) [49]	Prospective randomized open-label blinded-endpoint trial	Adults with persistent AF undergoing cardioversion; mostly no or minimal structural heart disease; small CAD subgroup	635 randomized	Efficacy: Flecainide was superior to no antiarrhythmic therapy at 4 weeks, with Kaplan–Meier survival free from persistent AF of 70.2% vs. 52.5% (*p* = 0.016). Long-term flecainide was more effective than short-term therapy after cardioversion. Safety: Serious adverse events were infrequent and similar across groups; among patients with CAD, events were not increased (1/37 [3%] vs. 9/583 [2%]; *p* = 0.462).
Ashraf et al. [46]	Retrospective cohort	Flecainide-treated patients with stable CAD evaluated by coronary angiography or myocardial perfusion imaging; included no/minimal, nonobstructive, and obstructive CAD; prior MI and LV dysfunction excluded	348	Safety: No increase in all-cause mortality across CAD strata over 10 years (*p* = 0.60), no excess ventricular arrhythmia burden (*p* = 0.25), no increased mortality with reversible perfusion defects (*p* = 0.14) or multivessel disease (*p* = 0.89). Efficacy: Not designed as a comparative efficacy study.
Kiani et al. [50]	Retrospective real-world cohort	AF patients treated with class Ic vs. class III antiarrhythmic drugs; CAD stratified as none, nonobstructive, or obstructive; excluded prior VT/VF, ICD, and nonrevascularized MI	3445 class Ic vs. 2216 class III	Safety: No excess mortality signal in selected nonobstructive CAD, but in obstructive CAD class Ic therapy was associated with worse event-free survival versus sotalol (HR 3.80, 95% CI 1.67–8.67; *p* = 0.002). Efficacy: Not primarily an efficacy study.
Burnham et al. [51]	Retrospective cohort with propensity matching	AF patients with stable CAD treated with flecainide vs. class III antiarrhythmic drugs; separate subgroup after PCI/CABG	Population 1: 1114 vs. 1114; Population 2: 150 vs. 1453	Safety: In matched stable CAD, flecainide was associated with lower VT (5.8% vs. 8.5%; *p* = 0.02), lower HF hospitalization (12.5% vs. 18.3%; *p* < 0.0001), and lower mortality (9.1% vs. 19.3%; *p* < 0.0001). In the PCI/CABG subgroup, outcomes numerically favored flecainide but were not statistically significant. Efficacy: Lower MACE with flecainide (22.9% vs. 36.6%; *p* < 0.0001).
Huang et al. [52]	Nationwide retrospective cohort	Adults with new-onset AF after PCI; class Ic users vs. nonusers in chronic coronary syndrome	3750	Safety: No increase in ventricular arrhythmia (asHR 0.89, 95% CI 0.69–1.15; *p* = 0.37); lower all-cause mortality (asHR 0.61, 95% CI 0.57–0.66; *p* < 0.01). Efficacy: Lower MACE (asHR 0.64, 95% CI 0.59–0.68) and lower cerebrovascular events (asHR 0.81, 95% CI 0.66–0.99; *p* = 0.04).
Rillig et al. (EAST-AFNET 4 analysis) [48]	Post hoc analysis of randomized trial	Early AF patients receiving sodium channel blockers for rhythm control; included selected stable cardiovascular disease, including prior MI/CABG/PCI	689 SCB users among 1395 early rhythm-control patients; 41 had prior MI/CABG/PCI	Safety: Primary safety outcome was not increased and was numerically lower with sodium channel blocker therapy (2.9 vs. 4.2 per 100 patient-years; adjusted *p* = 0.015). Efficacy: Primary efficacy outcome occurred less often with sodium channel blocker therapy (3.0 vs. 4.9 per 100 patient-years; *p* < 0.001); sinus rhythm at 2 years in 88% vs. 82%.
Tsioufis et al. (FLECA-ED) [53]	Preliminary randomized multicenter study	Paroxysmal AF with CAD, no residual ischemia, and ejection fraction > 35%; IV flecainide vs. amiodarone in the emergency department	25 total (10 flecainide, 15 amiodarone)	Safety: No safety signal with flecainide in this preliminary cohort; the only significant adverse event occurred in the amiodarone arm. Efficacy: Faster cardioversion with flecainide (35 vs. 679 min; *p* < 0.001); 100% of flecainide-treated patients were discharged in sinus rhythm within 6 h, whereas 73% of amiodarone-treated patients required hospitalization.
Sangpornsuk et al. [54]	Retrospective cohort	Flecainide-treated arrhythmic patients with and without structural heart disease; structural heart disease included a small chronic coronary syndrome subgroup	336 total; 47 structural heart disease; 5 chronic coronary syndrome	Safety: VT/VF occurred in 4.2% of structural heart disease patients vs. 1.0% without structural heart disease (*p* = 0.17); structural heart disease was not independently associated with VT/VF (adjusted OR 4.8, 95% CI 0.6–38.44; *p* = 0.14). Importantly, among the 5 chronic coronary syndrome patients, no patient experienced VT/VF after flecainide. Deaths were non-arrhythmic.

Abbreviations: AF, atrial fibrillation; asHR, adjusted subdistribution hazard ratio; CABG, coronary artery bypass grafting; CAD, coronary artery disease; CI, confidence interval; EAST-AFNET 4, Early Treatment of Atrial Fibrillation for Stroke Prevention Trial 4; HF, heart failure; HR, hazard ratio; ICD, implantable cardioverter–defibrillator; MACE, major adverse cardiovascular event; PCI, percutaneous coronary intervention; SCB, sodium channel blocker; VT, ventricular tachycardia; VT/VF, ventricular tachycardia/ventricular fibrillation.

**Table 2:** Studies Evaluating the Efficacy and Safety of Flecainide in Valvular Heart Disease.

Study/Author	Study Design	Population Included	Number of Patients	Findings (Efficacy and Safety)
Ghosh et al. [57]	Prospective single-center study	Chronic rheumatic AF after successful balloon mitral valvotomy; excluded LV/RV dysfunction, significant CAD risk, severe pulmonary hypertension, LA > 60 mm, AF > 5 years	50	Efficacy: SR at discharge in 38/50 (76%); maintained in 30/38 (79%) at 1 year (60% overall). Better NYHA class and physical QoL in those maintaining SR. Safety: No major complications, systemic embolism, deaths, hospitalizations, or proarrhythmia; no significant PR/QRS/QTc change.
Tripathi et al. [58]	Prospective single-center study	Chronic rheumatic AF after mitral valve replacement; excluded LV/RV dysfunction, severe pulmonary hypertension, LA > 80 mm, AF > 5 years, CAD/risk factors	25	Efficacy: SR in 21/25 (84%) at discharge; maintained in 16/21 (76%) initial converters and 16/25 (64%) overall at 6 months. Better NYHA class, QoL, and LA strain in those maintaining SR. Safety: No thromboembolism, major bleeding, hospitalizations, or deaths; no significant overall PR/QRS/QTc change.
Aabel et al. [59]	Case series	High-risk arrhythmic mitral valve syndrome with ventricular arrhythmias refractory to beta-blockers; normal coronary angiogram and no other structural heart disease	7	Efficacy: NSVT reduced from 21 episodes/90 patient-months to 0/250 patient-months; PVC burden fell from 4.2% to 0.4%/24 h (*p* < 0.001). Safety: No serious adverse effects or worsening ventricular arrhythmia burden; QRS increased by 16 ms.

Abbreviations: AF, atrial fibrillation; CAD, coronary artery disease; LA, left atrial; LV, left ventricular; NSVT, nonsustained ventricular tachycardia; NYHA, New York Heart Association; PVC, premature ventricular complex; QoL, quality of life; QTc, corrected QT interval; RV, right ventricular; SR, sinus rhythm.

**Table 3:** Studies Evaluating the Efficacy and Safety of Flecainide in Nonischemic Cardiomyopathy.

Study/Author	Study Design	Population Included	Number of Patients	Findings (Efficacy and Safety)
Dilated cardiomyopathy				
Raad et al. [60]	Retrospective cohort	Patients with nonischemic cardiomyopathy and implantable cardioverter–defibrillators treated with class Ic agents for PVC suppression; ischemia excluded in all	34 total; 23 flecainide, 11 propafenone	Efficacy: PVC burden decreased from 20% ± 13% to 6% ± 7% (*p* < 0.001); LVEF improved from 33% ± 9% to 37% ± 10% (*p* = 0.01); biventricular pacing increased from 85% ± 9% to 93% ± 7% (*p* = 0.005). Safety: Sustained VT occurred in 2 patients on therapy vs. 9 in the prior 12 months; HF admissions fell from 3 to 2; no deaths occurred during follow-up.
Sherman et al. [61]	Retrospective real-world cohort	Patients with nonischemic cardiomyopathy treated with class Ic agents for atrial fibrillation or PVC suppression	210 total; 86 flecainide, 124 propafenone	Efficacy: Mostly descriptive; mean PVC burden decreased by 1.08% (*p* = 0.604). Safety: No sustained VT and no cardiac deaths during treatment; 9 patients (4.29%) had NSVT; 5 deaths (2.3%) occurred, all adjudicated as unrelated to antiarrhythmic therapy.
Hypertrophic cardiomyopathy				
Haruki et al. [56]	Retrospective cohort	Patients with obstructive hypertrophic cardiomyopathy treated with oral flecainide compared with disopyramide	15 flecainide; 33 disopyramide	Efficacy: LV pressure gradient fell from 79.8 ± 36.6 to 39.2 ± 36.7 mmHg (*p* = 0.003); percent reduction −47.9% ± 43.2%, comparable to disopyramide (*p* = 0.425). NYHA class improved (*p* = 0.021). Safety: No significant adverse side effects, no early discontinuation, and no need for myectomy or alcohol septal ablation in flecainide-treated patients.
Arrhythmogenic cardiomyopathy				
Gaine et al. [62]	Multicenter retrospective cohort	Patients with definite ARVC receiving flecainide; included genotype-positive and gene-elusive cases, with and without left ventricular involvement	191	Efficacy: PVC burden decreased from 2190 to 418 (*p* < 0.001); NSVT fell from 35.1% to 21.5% (*p* = 0.003). In patients with prior ventricular arrhythmia, yearly VA episodes fell from 1.1 [0.4–1.6] to 0 [0–0.3] episodes/year (*p* < 0.001). Safety: Flecainide was well tolerated; discontinuation rate 7.9%. Overall mortality was 1.6%, with no arrhythmic sudden cardiac death; findings were consistent even in patients with left ventricular involvement (34.6%).
Rolland et al. [63]	Retrospective single-center cohort	Patients with definite or borderline ARVC treated with flecainide plus beta-blockers for persistent symptomatic ventricular arrhythmias	100	Efficacy: PVC burden decreased from median 2370 [1572–3400] to 415 [97–730] (*p* < 0.0001); programmed ventricular stimulation positivity fell from 94% off treatment to 40% on treatment (*p* < 0.001). Safety: Sustained VA occurred in 22 patients during follow-up, corresponding to an event rate of 5% at 1 year and 25% at 5 years; no deaths, no Brugada ECG pattern, and no hemodynamic impairment were reported.
Ermakov et al. [64]	Retrospective case series	Patients with definite ARVC refractory to single-agent therapy and/or catheter ablation treated with flecainide plus sotalol or metoprolol	8	Efficacy: 6/8 achieved excellent arrhythmia control and remained free of major ventricular arrhythmias for a mean of 35.5 months. Safety: 2/8 had recurrent arrhythmias within 2 months and underwent repeat ablation; no major safety signal was emphasized in this small series.
PVC-induced cardiomyopathy				
Hyman et al. [65]	Retrospective cohort	Patients with suspected PVC-induced cardiomyopathy treated with flecainide or propafenone after failed ablation and/or prior antiarrhythmic therapy	20	Efficacy: PVC burden decreased from 36.2% ± 3.5% to 10.0% ± 2.4% (*p* < 0.001); LVEF improved from 37.4% ± 2.0% to 49.0% ± 1.9% (*p* < 0.001). Similar improvement was seen in patients with limited myocardial delayed enhancement. Safety: Over 3.8 ± 0.9 years, no sustained ventricular arrhythmias or sudden cardiac deaths occurred.
Mann et al. [66]	Family-based genotype–phenotype study	Members of a large kindred with p.R222Q SCN5A–related arrhythmic dilated cardiomyopathy, characterized by frequent multifocal PVCs and variable DCM	17 genotype-positive carriers; treatment data highlighted in affected family members	Efficacy: In affected carriers with DCM, sodium channel–blocking therapy with flecainide or amiodarone led to marked reduction in ventricular ectopy and recovery of LV systolic function; reverse remodeling occurred over about 6 months after therapy initiation. Safety: The study emphasized clinical benefit without a major flecainide-related proarrhythmic signal in treated carriers.
Hyman et al. [67]	Retrospective cohort	Patients with suspected PVC-induced cardiomyopathy treated with flecainide or propafenone after failed ablation and/or prior antiarrhythmic therapy	20	Efficacy: PVC burden decreased from 36.2% ± 3.5% to 10.0% ± 2.4% (*p* < 0.001); LVEF improved from 37.4% ± 2.0% to 49.0% ± 1.9% (*p* < 0.001). Similar improvement was seen in patients with limited myocardial delayed enhancement. Safety: Over 3.8 ± 0.9 years, no sustained ventricular arrhythmias or sudden cardiac deaths occurred.
Kotoulas et al. (UNIFLECA) [68]	Prospective single-arm, nonrandomized study	Adults with frequent idiopathic PVCs (>5% burden on 2 Holters) who declined or were ineligible for ablation; a subset had impaired LVEF consistent with PVC-induced cardiomyopathy	35 treated; 19 with 1-month outcome data	Efficacy: Flecainide produced a mean PVC burden reduction of 76.2% in the first month; 63.1% achieved a reduction > 80%. Symptomatic improvement occurred in 74%, complete symptom resolution in 25.8%, and functional improvement was observed in patients with baseline LVEF impairment. Safety: No patient had a QRS increase > 25%, and no major adverse effects were reported.

Abbreviations: ARVC, arrhythmogenic right ventricular cardiomyopathy; DCM, dilated cardiomyopathy; ECG, electrocardiogram; HF, heart failure; LV, left ventricular; LVEF, left ventricular ejection fraction; NSVT, nonsustained ventricular tachycardia; NYHA, New York Heart Association; PVC, premature ventricular complex; VA, ventricular arrhythmia; VT, ventricular tachycardia.

**Table 4:** Studies Evaluating the Efficacy and Safety of Flecainide in a Pediatric Population and Congenital Heart Disease.

Study/Author	Study Design	Population Included	Number of Patients	Findings (Efficacy and Safety)
Hauguel-Moreau et al. (AFLOAT) [70]	Prospective multicenter randomized open-label trial with blinded endpoint evaluation	Adults after patent foramen ovale closure randomized to flecainide for 3 months, flecainide for 6 months, or standard care	186	Efficacy: Flecainide did not reduce post-procedural atrial arrhythmia; the primary endpoint occurred in 26.8% of flecainide-treated patients vs. 25.4% with standard care. Safety: Atrial arrhythmia occurred in 28.5% overall during 6 months, mostly in the first month; the study did not identify a preventive benefit of flecainide in this setting.
Smeets et al. [71]	Retrospective case series	Pregnant women treated transplacentally for non-hydropic fetal tachycardia; digoxin first-line, with flecainide added for treatment failure	28 pregnancies; 18 required flecainide	Efficacy: Digoxin alone restored sinus rhythm in 9/28 (32%); after addition of flecainide, overall conversion increased to 26/28 (93%). Safety: Flecainide increased maternal side effects, mainly nausea, but these were manageable with dose reduction; median fetal-to-maternal flecainide ratio was 0.82.
Bertels et al. (ECTOPIC) [72]	Randomized open-label crossover trial	Children with frequent idiopathic premature ventricular contractions (>15% burden) and structurally normal hearts treated successively with flecainide and metoprolol	19	Efficacy: Mean PVC reduction was 10.6 percentage points with flecainide vs. 2.4 percentage points with metoprolol; between-treatment difference 8.2 percentage points (*p* = 0.031). A reduction to PVC burden < 5% occurred in 9/18 on flecainide vs. 1/17 on metoprolol. Safety: No major safety signal was highlighted in this small pediatric crossover study.
Strizek et al. [73]	Retrospective observational study	Fetuses with supraventricular tachyarrhythmia with and without hydrops treated transplacentally; flecainide, digoxin + flecainide, or digoxin	46 fetuses; 42 treated; 28 flecainide monotherapy, 4 digoxin + flecainide	Efficacy: Among fetuses treated with flecainide as first-line therapy (monotherapy or combination), 26/32 (81.2%) converted to sinus rhythm; with flecainide monotherapy, conversion occurred in 72.2% of hydropic and 90% of nonhydropic fetuses, with median time to sinus rhythm 3 days. Safety: Maternal side effects were rare; one asymptomatic mother developed a Brugada ECG pattern that resolved after flecainide discontinuation.
Cunningham et al. [69]	Retrospective 2-center cohort	Pediatric patients receiving flecainide for supraventricular or ventricular arrhythmias; included children with CHD, cardiomyopathy, and normal hearts	175 total; 20 CHD, 2 cardiomyopathy	Efficacy: Arrhythmia control was achieved in 90% of CHD patients vs. 77% in those with normal hearts. Safety: Flecainide-associated cardiac dysfunction requiring discontinuation occurred in 2 patients (1%), proarrhythmia in 3 (2%), and no cardiac arrests occurred; fewer than 3% discontinued because of flecainide-related adverse events.
Moffett et al. [74]	Retrospective multicenter administrative cohort	Children with CHD or cardiomyopathy receiving enteral antiarrhythmic therapy for supraventricular arrhythmias	3544 total; 229 received flecainide	Efficacy: Primarily a utilization/safety study; flecainide use rose from 4.6% in 2004 to 8.7% in 2011. Safety: Cardiac arrest occurred in 3.0% and overall mortality in 4.3% of flecainide-treated patients, but flecainide was not associated with higher cardiac arrest or death than other antiarrhythmics on multivariable analysis; mortality in cardiomyopathy was 2.9%, and no patient with single-ventricle physiology died.

Abbreviations: CHD, congenital heart disease; PVC, premature ventricular complex.

## Data Availability

All data generated in this research are included within the article.
